# Toll-Like Receptor 4 Promotes NO Synthesis by Upregulating GCHI Expression under Oxidative Stress Conditions in Sheep Monocytes/Macrophages

**DOI:** 10.1155/2015/359315

**Published:** 2015-10-20

**Authors:** Shoulong Deng, Kun Yu, Baolu Zhang, Yuchang Yao, Zhixian Wang, Jinlong Zhang, Xiaosheng Zhang, Guoshi Liu, Ning Li, Yixun Liu, Zhengxing Lian

**Affiliations:** ^1^State Key Laboratory of Reproductive Biology, Institute of Zoology, Chinese Academy of Sciences, Beijing 100101, China; ^2^Laboratory of Animal Genetics and Breeding, College of Animal Science and Technology, China Agricultural University, Beijing 100193, China; ^3^National Key Laboratory of Agrobiotechnology, College of Biological Sciences, China Agricultural University, Beijing 100193, China; ^4^College of Animal Science and Technology, Northeast Agricultural University, Harbin 150030, China; ^5^Tianjin Institute of Animal Sciences, Tianjin 300112, China

## Abstract

Many groups of Gram-negative bacteria cause diseases that are harmful to sheep. Toll-like receptor 4 (TLR4), which is critical for detecting Gram-negative bacteria by the innate immune system, is activated by lipopolysaccharide (LPS) to initiate inflammatory responses and oxidative stress. Oxidation intermediates are essential activators of oxidative stress, as low levels of free radicals form a stressful oxidative environment that can clear invading pathogens. NO is an oxidation intermediate and its generation is regulated by nitric oxide synthase (iNOS). Guanosine triphosphate cyclohydrolase (GCHI) is the rate-limiting enzyme for tetrahydrobiopterin (BH4) synthesis, which is essential for the production of inducible iNOS. Previously, we made vectors to overexpress the sheep *TLR4* gene. Herein, first generation (G1) of transgenic sheep was stimulated with LPS *in vivo* and *in vitro*, and oxidative stress and GCHI expression were investigated. Oxidative injury caused by TLR4 overexpression was tightly regulated in tissues. However, the transgenic (Tg) group still secreted nitric oxide (NO) when an iNOS inhibitor was added. Furthermore, GCHI expression remained upregulated in both serum and monocytes/macrophages. Thus, overexpression of TLR4 in transgenic sheep might accelerate the clearance of invading microbes through NO generation following LPS stimulation. Additionally, TLR4 overexpression also enhances GCHI activation.

## 1. Introduction

In both host defense and inflammation, monocytes/macrophages are the most prominent immune cell type activated by lipopolysaccharide (LPS) to release various pro- and anti-inflammatory mediators. Toll-like receptor 4 (TLR4) interacts with several proteins to form the myeloid differentiation factor 2 (MD2)/CD14/TLR4 complex that binds to LPS [1]. TLR4 then recruits downstream adaptors to activate both the myeloid differentiation primary response gene 88 (MyD88) and TIR-domain-containing adaptor-inducing interferon-*β*- (TRIF-) dependent pathways, which function through nuclear factor-*κ*B- (NF-*κ*B-) associated signaling events [2]. The actions of these proteins trigger the release of oxidation intermediates, including reactive oxygen species (ROS), reactive nitrogen species (RNS), and cytokines [3]. Apart from the inflammatory reaction, oxidation intermediates are also involved in many pathological processes, such as insulin resistance and type 2 diabetes [4]. They are extremely important for the intracellular environment. To maintain physiological homeostasis, ROS are maintained at a certain level to induce enough oxidative stress to eliminate pathogenic microorganisms. However, large amounts of oxidation intermediates and derivatives can both destroy bacterial membranes and cause host tissue damage.

ROS are mainly produced by nicotinamide adenine dinucleotide phosphate (NADPH) oxidase. NADPH oxidase is a multisubunit enzyme complex. When electrons are transported across membranes, the membranes are depolarized. These changes lead to compensatory ion flux. NADPH oxidase has specialized domains for the dismutation of O_2_^−^ and H_2_O_2_ production [5]. NAPDH oxidase activation is critically regulated by cytokines produced in response to activation of the TLR4 pathway. Interleukin-1 receptor-associated kinase-4 (IRAK-4), the regulatory kinase, which is downstream of TLR4 signaling, regulates NADPH oxidase through the phosphorylation of p47^phox^ [6]. Tumor necrosis factor-alpha (TNF-*α*) enhances the activity of NADPH oxidase through the NF-*κ*B pathway by upregulating mRNA expression levels of transcripts that encode gp91^phox^ [7]. NF-*κ*B activation is mediated by direct interactions of TLR4 with Nox4 and Nox2, two subunits of NAPDH oxidase [8, 9].

NO is the major component of RNS. In mononuclear macrophages, inducible nitric oxide synthase (iNOS) is the rate-limiting enzyme for NO synthesis. Along with NAPDH, O_2_, and tetrahydrobiopterin (BH4), NO is synthesized from L-arginine by the enzyme iNOS. In the inflammatory response, transcription of iNOS is regulated by the NF-*κ*B and MAPK pathways. Increasing levels of downstream factors, such as interleukin-1 (IL-1), TNF-*α*, and interferon-gamma (IFN-*γ*), result in increased iNOS expression [10].

Another essential enzyme for iNOS* de novo* biosynthesis is BH4. BH4 deficiency causes a reduction in iNOS uncoupling. The rate-limiting enzyme for BH4 is GTP cyclohydrolase, which is encoded by GCHI [11]. GCHI is important in iNOS activation and is closely linked to various pathological processes, such as vasofunctional disturbances and dyskinesia [12].

Innate immunity is the major type of defense against Gram-negative bacterial infection. TLR4 participates in innate immunity in various ways, not only in resistance to Gram-negative bacterial infections, but also in many autoimmune and inflammatory disease settings, including atherosclerosis, diabetes mellitus, cancer, and rheumatoid arthritis. Previous studies have shown that the susceptibility or sensitivity of TLR4-mutant cells to LPS is lower than that of wild-type cells [13, 14]. Furthermore, TLR4-deficient mice stimulated with LPS cannot secrete IL-1 or IL-12 [15] and also show reduced expression of IL-6 [16]. However, TLR4 overexpression in mice results in increased disease resistance [17]. We previously reported the generation of TLR4-transgenic sheep [18]. The effect of bacteria on the sensitivity of transgenic animals to disease resistance is dependent on transgene copy number [19]. Notably, excessive inflammation and oxidative stress can cause tissue damage [20]. To better understand the biological basis for a role of overexpressed TLR4 in the immune response and oxidative stress, we isolated monocytes/macrophages from the peripheral blood of first-generation (G1) transgenic sheep with two transgene copies and stimulated them with LPS. Immunoactivity and oxidative damage were investigated. Furthermore, we added iNOS and NADPH oxidase inhibitors to LPS-stimulated monocytes/macrophages to study the relationship between TLR4 and oxidative stress. Herein, we first demonstrate that overexpression of TLR4 promotes NO synthesis by upregulating GCHI expression under oxidative stress conditions in sheep monocytes/macrophages. Innate immune responses and oxidative stress in TLR4-transgenic sheep were tightly regulated.

## 2. Materials and Methods

### 2.1. Ethics Statement

Artificial insemination, intradermic injection, and blood collection were performed at the experimental station of the China Agricultural University, and the whole procedure was carried out in strict accordance with the protocol approved by the Animal Welfare Committee of China Agricultural University (Permit Number: XK662).

### 2.2. Screening of Transgenic Sheep Overexpressing TLR4

Genomic DNA from TLR4-transgenic sheep was extracted from sperm and a 623-bp fragment was amplified with the following pair of primers: Forward, TAC GGT AAA CTG CCC ACT TG; Reverse, ACC TGG AGA AGT TAT GGC TG. In mating season, ewes of natural estrus were inseminated with sperm from positive sheep. The presence of a transgene in offspring was analyzed by Southern blotting. We used polymerase chain reaction (PCR) to generate specific digoxigenin-labeled probes (Roche Diagnostics, Mannheim, Germany) using the primer sequences indicated above. Genomic DNA was isolated from ear tissue and digested with VspI and SmaI (NEB, Beverly, MA, USA).

Mononuclear cells were isolated from the peripheral blood of G1 transgenic sheep using sheep lymphocyte separation medium (TBD, Tianjin, China). Total RNA was extracted. The mRNA abundance of the* TLR4* gene was measured by real-time PCR, using a SYBR Green kit (Tiangen, China) with the following primers: TLR4-forward, CTG AAT CTCTAC AAA ATC CC; TLR4-reverse, CTT AAT TTC GCA TCT GGA TA; *β*-actin-forward, AGA TGT GGA TCA GCA AGC AG; *β*-actin-reverse, CCA ATC TCA TCT CGT TTT CTG. TLR4 protein levels were measured by enzyme-linked immunosorbent assay (ELISA; Shanghai Xin Le, Shanghai, China).

Peripheral blood was collected for routine blood and serum biochemical parameter testing at 120 days. Testing included analysis of red blood cells, white blood cells, hemoglobin, albumin, globulin, alanine aminotransferase, aspartate aminotransferase, glucose, blood urea nitrogen, and serum total protein concentrations.

### 2.3. Detection of Acute Inflammatory Responses in G1 Transgenic Sheep* In Vivo*

The ears of five 3-month-old transgene-positive sheep were injected with 100 *μ*L 3 mg/mL LPS (Sigma-Aldrich, St. Louis, MO, USA); then tissues were collected at 1, 8, and 48 h. Samples were fixed with 4% paraformaldehyde and embedded in paraffin. Hematoxylin and eosin staining was used to investigate the inflammatory response and immunohistochemistry was used to detect MD2 protein expression (Abcam, Cambridge, UK). Peripheral blood was also collected at 1, 4, 8, and 48 h. RNA was isolated from mononuclear cells and real-time PCR was used to detect the expression of* TLR4*,* CD14*, and* NF-κB*. Primer sequences were as follows: TLR4: as above; CD14-forward, ATA TCT AGC ACT ACG CAA CGC; CD14-reverse, CTT GGT CGG CAG TCC TTT; NF-*κ*B-forward, TTC TCC AAA TGG CTG AAG GTA; NF-*κ*B-reverse, TTG TTT GAG GGC CAT AAG GAT. ELISA kits were used to detect levels of TLR4, GCHI, and various cytokines, including IFN-*γ*, TNF-*α*, IL-6, IL-8, IL-12, and IL-10 in serum (Shanghai Xin Le).

### 2.4. Oxidative Stress in LPS-Stimulated Sheep Mononuclear Cells

Sheep peripheral blood mononuclear cells were isolated and cultured in RM1640 (Gibco, Grand Island, NY, USA) medium containing 10% fetal bovine serum (Gibco) for 48 h. After stimulation with LPS (1 *μ*g/mL), cell suspensions were collected at 0, 1, 8, and 48 h. The activities of iNOS and NADPH oxidase, as well as the ROS, RNS, and malonaldehyde (MDA) contents of the cells, were examined by spectrophotometry (Nanjing Jiancheng Bioengineering Institute, Nanjing, China). The activity of GCHI was examined by an enzymatic method (Nanjing Jiancheng Bioengineering Institute). After 1 h stimulation with LPS, 4% paraformaldehyde was used to fix cells; then immunofluorescence was used to detect protein oxidation products. Thioredoxin- (TRX-) TRITC (Abcam) and TLR4-FITC (Abcam) were used to detect changes in protein expression, and DAPI was used to counterstain nuclei. Confocal microscopy was used to detect staining results.

NADPH oxidase and iNOS inhibitors were added to monocytes/macrophages during stimulation with 1 *μ*g/mL LPS to study the relationship between TLR4 and oxidative damage. Apocynin (Sigma-Aldrich) is an inhibitor of NADPH oxidase and inhibits the production of O_2_^−^. Nitro-L-arginine (L-NNA) (Sigma-Aldrich) is an inhibitor of iNOS and inhibits the production of NO. Inhibitors were used at concentrations of 10 and 20 mmol/L. A TLR4 inhibitor, anti-TLR4 antibody, was added to the Tg group culture media at a dilution of 1 : 500. After 1 h, cell suspensions were collected. The NO, O_2_^−^, and MDA concentrations were determined by spectrophotometry according to the manufacturer's instructions (Nanjing Jiancheng Bioengineering Institute).

### 2.5. Statistical Analyses

All experiments repeated 3 times. All data were subjected to analysis of variance using the GLM procedures of Statistical Analysis System (SAS Institute, Cary, NC, USA). All data were expressed as mean ± SEM. Differences were considered to be significant when *P* < 0.05.

## 3. Results

### 3.1. Overexpression of TLR4 in G1 Transgenic Sheep

**Figure 1:**
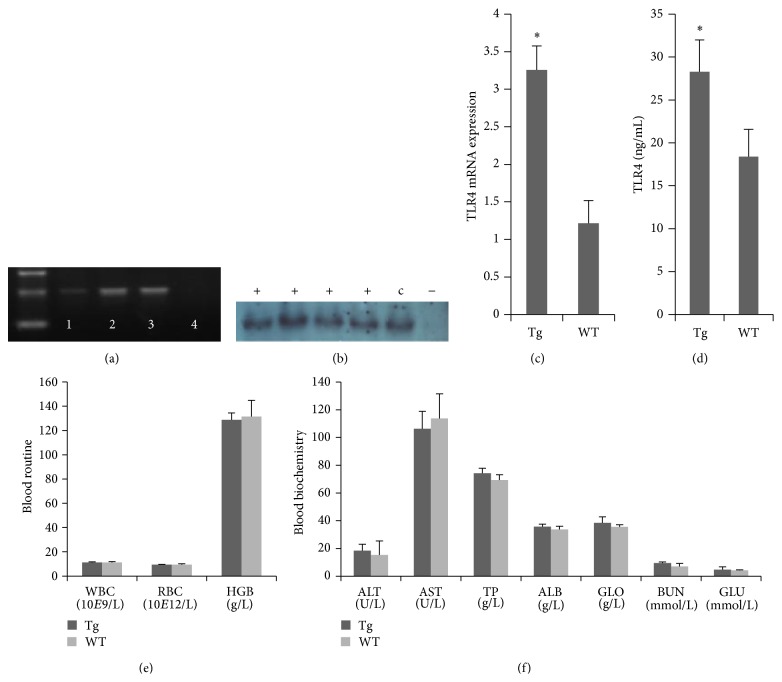
Overexpression of TLR4 in G1 transgenic sheep. (a) Transgene-positive sperm from transgenic sheep were detected by PCR. Lanes: (1) TLR4-positive ovine sperm genomic DNA, (2) TLR4-positive ovine ear genomic DNA, (3) expression vector (p3S-LoxP-TLR4), and (4) negative control. (b) Southern blot analysis of the TLR4 transgene insert in transgenic G1 lambs. “+”: TLR4-transgenic G1 lambs, “−”: negative control, and “c”: TLR4-transgenic sheep. TLR4 expression and translation in mononuclear cells from G1 transgenic lambs were detected by real-time quantitative PCR and ELISA; more TLR4 was detected in transgenic lambs (c) and (d). Blood routine (e) and blood biochemistry (f) examinations were carried out on TLR4-transgenic sheep. There were no statistically significant differences between G1 and WT lambs. Tg: TLR4-transgenic G1 lambs, WT: wild-type. The results are expressed as mean ± SE; ^*^ *P* < 0.05 in Tg versus WT groups.

We previously generated transgenic sheep using the microinjection technique. Transgenic sheep had two TLR4 copies integrated into germ cells, as determined by PCR (Figure 1(a)). Artificial insemination was then used for the propagation of sheep. Southern blot detection showed that the G1 sheep were positive for exogenous TLR4 (Figure 1(b)). Real-time PCR and ELISA were performed to determine the expression levels of TLR4. The expression of TLR4 in Tg sheep was significantly higher than in WT sheep (Figures 1(c) and 1(d)). There were no statistically significant differences in the analysis of the routine blood counts or biochemical serum parameters between transgenic and WT sheep at 120 days (Figures 1(e) and 1(f)).

### 3.2. TLR4 Overexpression in G1 Tg Sheep Triggered Rapid Neutrophil Infiltration

**Figure 2:**
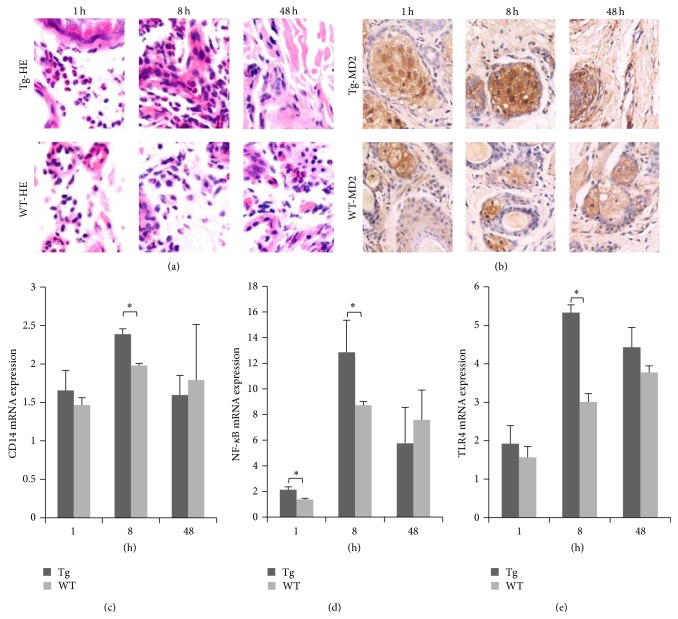
LPS stimulation triggered rapid infiltration of neutrophils in transgenic sheep. (a) Pathological changes were examined microscopically (hematoxylin and eosin staining, ×400). (b) MD2 expression was detected by immunohistochemistry (×200). (c) CD14, (d) NF-*κ*B, and (e) TLR4 transcription in mononuclear cells from G1 transgenic lambs were detected by real-time quantitative PCR. Tg: transgenic sheep, WT: wild-type. The results are expressed as mean ± SE; ^*^ *P* < 0.05 in Tg versus WT groups.

LPS was injected into the ears of transgenic sheep and the resulting inflammatory infiltrate was observed under a light microscope after hematoxylin and eosin staining (Figure 2(a)). In the Tg group, many segmented neutrophils infiltrated the dermis at 1 h, and after 8 h more inflammatory cells, including many neutrophils, had infiltrated the dermis and many erythrocytes had infiltrated the connective tissues. Fewer inflammatory cells were observed in Tg animals at 48 h after stimulation. This finding suggested that the inflammatory reaction in Tg animals was negatively regulated by a feedback loop in order to avoid inflammatory injury. However, no significant lesions were observed after 48 h. In the WT group, dermis bleeding also occurred with inflammatory cell infiltration at 1 h. After 8 h, many erythrocytes were spread across the surface of the skin and between connective tissues, and inflammatory cells—including many neutrophils—had infiltrated around the blood vessels. Few infiltrating inflammatory cells were evident after 48 h. Immunohistochemistry was used to observe MD2 protein expression. MD2-positive tissues showed claybank, which was primarily expressed by sebaceous gland cells and infiltrating inflammatory cells (Figure 2(b)).

Real-time PCR showed that CD14 and TLR4 transcription reached a peak at 8 h. By contrast, TLR4 transcription continued to rise in the WT group (Figures 2(c) and 2(e)). These above findings showed that, at 8 h, the inflammatory response of the Tg group was stronger than that of the WT group. After 48 h, the inflammatory response of the Tg group had finished, whereas the inflammatory response of the WT group remained in progress, indicating that Tg sheep can launch a more rapid inflammatory reaction. Transcription of the TLR4 downstream factor NF-*κ*B was also significantly higher in the Tg group than in the WT group at 1 and 8 h (Figure 2(d)). These results suggest overexpression of* TLR4* gene could trigger rapid neutrophil infiltration.

### 3.3. Overexpression of TLR4 Enhanced Oxidative Stress in Sheep Monocytes/Macrophages

**Figure 3:**
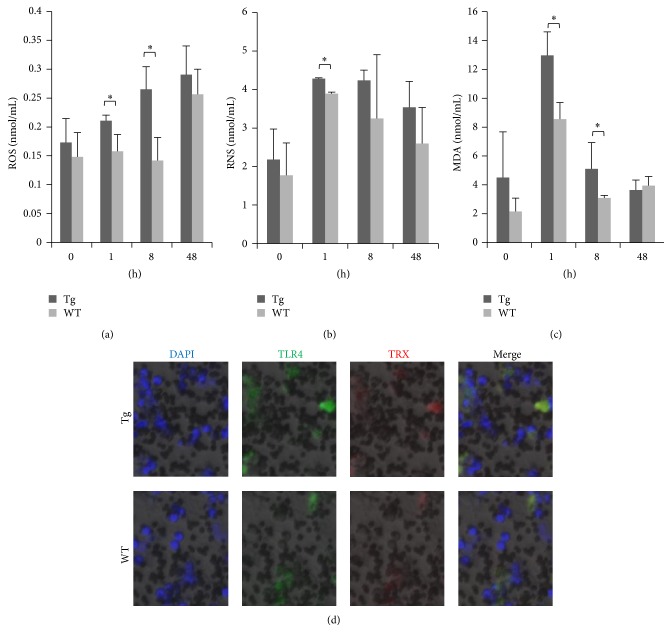
Overexpression of TLR4 enhances oxidative stress in monocytes/macrophages. Expression patterns of ROS, RNS, and MDA after stimulation with 1 *μ*g/mL LPS ((a), (b), and (c), resp.). (d) TLR4 and TRX protein expression levels were detected by immunofluorescence (×200), DAPI (blue), TLR4-FITC (green), and TRX (red). Tg: transgenic sheep, WT: wild-type. Results were expressed as mean ± SE; ^*^ *P* < 0.05 in Tg versus WT groups.

After stimulation with LPS, ROS expression increased in the Tg group, with significant differences compared with the WT group evident at 1 and 8 h (*P* < 0.05). By contrast, the expression of ROS was maximal at 48 h in the WT group (Figure 3(a)). Compared with the WT group, the expression of RNS was also significantly higher at 1 h in the Tg group (*P* < 0.05); however, there was no significant difference between groups at 48 h (Figure 3(b)). In the Tg group, the release of ROS/RNS was increased, indicating that overexpression of TLR4 could upregulate the expression of ROS/RNS, inducing a stronger oxidative stress response in monocytes/macrophages. The expression of MDA also showed a significant difference at 1 and 8 h (*P* < 0.05) between the Tg and WT groups, indicating that overexpression of TLR4 in monocytes/macrophages enhanced oxidative damage. However, at 48 h, MDA expression returned to normal levels in the Tg group (Figure 3(c)). The expression of TRX protein in monocytes/macrophages 1 h after stimulation with LPS was assessed by immunofluorescence. Compared with the WT group, expression of both TLR4 and TRX protein in the Tg group was higher and the degree of oxidative damage was stronger (Figure 3(d)).

### 3.4. TLR4-Mediated Oxidative Stress Triggered by Monocyte/Macrophage NO Secretion

**Figure 4:**
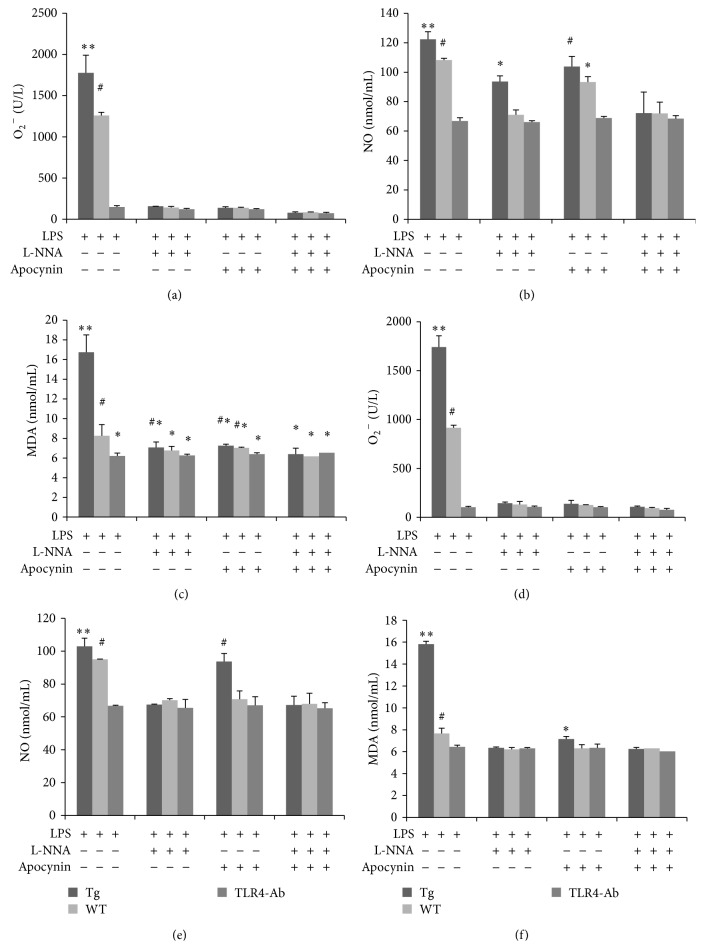
Overexpression of TLR4 induced oxidative stress via the secretion of NO by monocytes/macrophages. Levels of O_2_^−^, NO, and MDA were examined in monocytes/macrophages after LPS stimulation. Cells were treated with various combinations of the inhibitors apocynin (10 mmol/L) and L-NNA (10 mmol/L; (a), (b), and (c)). Similar patterns were observed when inhibitor concentrations were raised to 20 mmol/L ((d), (e), and (f)). Tg: transgenic sheep, WT: wild-type, and TLR4-Ab: anti-TLR4 antibody. The results are expressed as mean ± SE. ^∗, ∗∗, #^Values were found to be significantly different between groups (*P* < 0.05).

*In vivo*, NO and O_2_^−^ are mainly released by mononuclear macrophages. TLR4 regulates the expression of NADPH oxidase, iNOS, and apocynin, which is an inhibitor of NADPH oxidase that can inhibit the release of O_2_^−^. Furthermore, L-NNA, which is an inhibitor of iNOS, can inhibit NO production. Monocytes/macrophages were stimulated with 1 *μ*g/mL LPS, and 10 mmol/L of apocynin and/or L-NNA were added to inhibit the expression of NADPH oxidase and iNOS, respectively. The cellular content of O_2_^−^, NO, and MDA was then determined (Figures 4(a), 4(b), and 4(c)). Our results showed that the expression of NO increased in the LPS-stimulated Tg group treated with 10 mmol/L L-NNA, whereas the expression of O_2_^−^ was reduced. Expression of NADPH oxidase was inhibited in the LPS-stimulated Tg group by 10 mmol/L apocynin, while O_2_^−^ concentrations were also reduced. For expression of NO, the difference in the Tg group with inhibitor was more significant than that in the Tg group without inhibitor (*P* < 0.05). Then, 20 mmol/L L-NNA was added (Figures 4(d), 4(e), and 4(f)) and this inhibited the expression of iNOS. In the Tg group, expression of NO was also inhibited, the content of O_2_^−^ was reduced, and there was no strong oxidative damage. Although 20 mmol/L apocynin inhibited the expression of NADPH oxidase and the content of O_2_^−^ was reduced, NO was still secreted by the Tg group. Compared with controls, expression of MDA in the Tg group was significantly different (*P* < 0.05). These data indicated that TLR4 induced oxidative stress by promoting the release of NO by monocytes/macrophages.

### 3.5. TLR4 Upregulation of GCHI Activation in Sheep Monocytes/Macrophages and Serum

**Figure 5:**
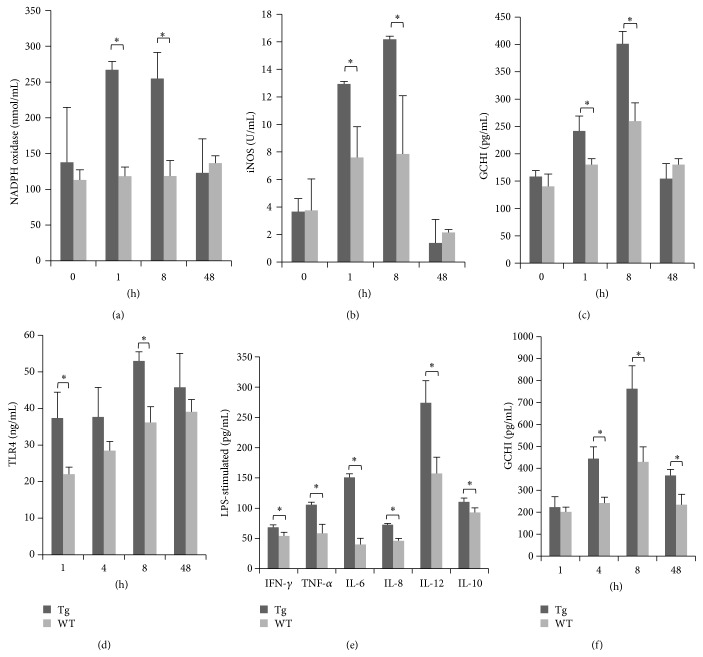
TLR4 enhanced GCHI activity in monocytes/macrophages and serum. The activities of NADPH oxidase, iNOS, and GCHI in monocytes/macrophages were examined after LPS stimulation ((a), (b), and (c), resp.). (d) Levels of TLR4 in the serum after LPS stimulation were measured by ELISA. (e) The effects of LPS stimulation on the expression of immune factors (TNF-*α*, IFN-*γ*, IL-6, IL-8, IL-12, and IL-10) in serum. (f) GCHI expression in serum was detected by ELISA. Tg: transgenic sheep, WT: wild-type. Data are expressed as mean ± SE; ^*^ *P* < 0.05 in Tg versus WT groups.

In macrophages of the Tg group stimulated with LPS, expression of NADPH oxidase and iNOS was significantly greater than that of the WT group at 1 and 8 h (*P* < 0.05) but returned to a normal level at 48 h (Figures 5(a) and 5(b)). This finding indicates that TLR4 could regulate the expression of NADPH oxidase and iNOS. GCHI plays an important role in the regulation of iNOS expression. The expression of GCHI in the Tg group was also significantly higher at 1 and 8 h (*P* < 0.05) and returned to a normal level at 48 h, similar to iNOS (Figure 5(c)). These findings indicated that TLR4 enhances the activity of iNOS by upregulating GCHI to promote synthesis and secretion. In serum, during an acute inflammatory reaction, expression of TLR4 protein was significantly different between groups at 1 and 8 h (*P* < 0.05). Furthermore, the Tg group showed attenuated TLR4 expression at 48 h, while TLR4 expression in the WT group continued to increase (Figure 5(d)). We detected proinflammatory cytokines downstream of TLR4 at various time points, IFN-*γ* (1 h), TNF-*α* (1 h), IL-6 (8 h), IL-12 (8 h), and IL-8 (8 h), and anti-inflammatory cytokines IL-10 (48 h), and all showed significant differences between the Tg and WT group (*P* < 0.05; Figure 5(e)). This finding indicates that overexpression of the* TLR4* gene in sheep can promote the expression of downstream inflammatory cytokines, inducing rapid inflammatory reactions. Expression levels of GCHI protein in serum of the Tg group were significantly higher than those in the WT group at 4, 8, and 48 h (*P* < 0.05; Figure 5(f)). This finding indicates that TLR4 regulates the expression of GCHI through downstream inflammatory cytokines.

## 4. Discussion

When pathogenic microorganisms invade animals, the TLR4 signaling pathway is activated and triggers a cascade of reactions to promote the production and release of inflammatory cytokines, inducing the chemotactic aggregation of granulocytes and macrophages, increased capillary permeability, and lymphocyte infiltration. The TLR4 signal transduction pathway is involved in many distinct diseases [21–23]. Current research on TLR4 signaling is concentrated in the areas of infectious disease, pulmonary infections, cancer, type 2 diabetes, and sepsis [24, 25]. Activation of TLR4 is beneficial for the elimination of exogenous pathogens. A TLR4 activator, BCG (Bacillus Calmette, Guérin), has been used in bladder cancer treatment for its ability to trigger the production of cytokines and enhance immune responses [26]. TLR4 induced large amounts of cytokine release, which is also observed in insulin resistance. Through those preinflammatory kinases and ROS, TLR4 directly represses insulin action [27]. This current study reports the generation of TLR4-transgenic sheep with stable transgene transmission. We have shown that TLR4 was overexpressed at the RNA level and protein levels. Overexpression of TLR4 did not show any harmful effects on animal health. The result of an acute inflammatory reaction triggered by LPS indicates that overexpression of the* TLR4* gene induced the rapid infiltration of neutrophils. Thus, TLR4 can upregulate the expression of cytokines, such as TNF-*α*, IFN-*γ*, IL-6, IL-8, IL-12, and IL-10, thereby increasing resistance to pathogen invasion and infection.

As an important immune receptor, TLR4 plays a central role in oxidative stress. Research of oxidative lung injury in mice with targeted deletions of multiple inflammatory, immune, and antioxidant genes indicated that TLR4 is an important candidate gene related to oxidative-stimulated inflammation [28, 29]. TLR4 can trigger transcription of the iNOS gene and promote production of NO. The main function of NO is clearing pathogenic bacteria by producing peroxidase and superoxide radicals [30]. ROS are mainly induced by NADPH oxidase, whose activity is regulated by TLR4 through the composition of the NADPH multisubunit enzyme complex. ROS could oxidize the iNOS cofactor BH4, reduce the expression of BH4, and lead to uncoupling of iNOS to form more O_2_^−^. ROS are produced under conditions of the uncoupling of NADPH oxidase and mitochondria when the body is stimulated by oxidative stress. ROS can induce NF-*κ*B activation and upregulate the cytokine-induced iNOS gene, resulting in excessive release of NO [31]. When the levels of NO are higher than those of ROS, NO can clear ROS. Currently, NOS uncoupling is thought to play a major role in the induction of oxidative stress [32, 33]. In the present study, NO was expressed in TLR4-overexpressing cells stimulated with LPS, even after NADPH oxidase was added. This finding indicates that, in sheep mononuclear macrophages, TLR4 promotes the secretion of NO by regulating iNOS expression. Similarly, when NO and O_2_^−^ production were investigated in NOS-transfected cells, an iNOS-specific inhibitor could drastically reduce the production of O_2_^−^ [34]. Our results are in accordance with this, as L-NNA inhibited the expression of iNOS and reduced the expression of O_2_^−^. TLR4 overexpression could also protect against oxidative stress [35], and NO could clear O_2_^−^ [36]. TLR4 induces the expression of NO by upregulating iNOS, and NO induces expression of many genes that can reduce oxidative stress and help cells resist injury [37]. The present study suggests that TLR4-overexpressing sheep could reduce oxidative injury more efficiently.

GCHI is the rate-limiting enzyme in BH4 biosynthesis. BH4 is an important cofactor for NOS. When the production of BH4 is limited, coupling of O_2_ and L-arginine are reduced, resulting in increased O_2_^−^ catalyzed by NOS and no increase in NO. Recent studies showed that reductions in BH4 are linked to hypertension, arteriosclerosis, diabetes mellitus, cardiac hypertrophy, and myocardial ischemia [38, 39]. Sakai et al. found that a phosphatidylinositol 3-kinase (PI3K) inhibitor could inhibit the synthesis of NO in macrophages stimulated by LPS, while synthesis of NO and the activation of GCHI could both be inhibited in PI3K-deficient macrophages [40]. After stimulation with cytokines (IFN-*γ* or TNF-*α*) or LPS, the expression of iNOS increased significantly, resulting in increased synthesis of NO, which had a direct relationship with the increased BH4 synthesis caused by the activated GCHI gene [41]. A study of GCHI-transgenic mice stimulated with LPS showed that the expression of renal iNOS and NO content increased dramatically [42]. A GCHI inhibitor could significantly inhibit the production of NO induced by LPS [43]. LPS binds to macrophage TLR4 and activates downstream signaling pathways, including NF-*κ*B, mitogen-activated protein kinase (MAPK), and PI3K. These signaling pathways can promote macrophages to release cytokines, including IFN-*γ* and TNF-*α*. We used LPS to stimulate transgenic sheep overexpressing TLR4 as a model of acute inflammation. TLR4 overexpression* in vivo* could enhance expression of downstream inflammatory factors and GCHI. This finding indicates that TLR4 regulates GCHI expression through downstream inflammatory factors. In endothelial cells, GCHI overexpression could enhance 10-fold the production of BH4, accompanied with significant increases in NO [44]. Overexpression of GCHI in transgenic mouse endothelial cells could reduce O_2_^−^ significantly and decrease production of superoxide, while bioavailability of NO was well maintained in GCHI-transgenic mice [45]. Our results show that TLR4 could upregulate the expression of NADPH oxidase and iNOS in mononuclear macrophages. The Tg cells stimulated with LPS plus an iNOS inhibitor still secreted NO, and the expression of GCHI was upregulated. These findings indicate that TLR4 enhances the activity of iNOS by upregulating GCHI, which then promotes the synthesis and secretion of NO.

In summary, these experiments document that the novel overexpression of TLR4 in transgenic sheep enhanced oxidative stress and that TLR4-induced oxidative stress was caused by NO. TLR4 and its downstream signaling pathways play important roles in the activation of GCHI expression. This study provides valuable insights into oxidative disease caused by Gram-negative bacterial infection in sheep.

## References

[1] Hoareau L., Bencharif K., Rondeau P. (2010). Signaling pathways involved in LPS induced TNFalpha production in human adipocytes. *Journal of Inflammation*.

[2] Tse K.-H., Chow K. B. S., Leung W. K., Wong Y. H., Wise H. (2014). Lipopolysaccharide differentially modulates expression of cytokines and cyclooxygenases in dorsal root ganglion cells via Toll-like receptor-4 dependent pathways. *Neuroscience*.

[3] Ryan K. A., Smith M. F., Sanders M. K., Ernst P. B. (2004). Reactive oxygen and nitrogen species differentially regulate Toll-like receptor 4-mediated activation of NF-kappa B and interleukin-8 expression. *Infection and Immunity*.

[4] Uchimura K., Hayata M., Mizumoto T. (2014). The serine protease prostasin regulates hepatic insulin sensitivity by modulating TLR4 signalling. *Nature Communications*.

[5] Zaitseva O. O., Polezhaeva T. V., Khudyakov A. N. (2013). Influence of pectins on NADPH oxidase and phagocytic activity of neutrophils during cryopreservation. *Cryo-Letters*.

[6] Pacquelet S., Johnson J. L., Ellis B. A. (2007). Cross-talk between IRAK-4 and the NADPH oxidase. *Biochemical Journal*.

[7] Gauss K. A., Nelson-Overton L. K., Siemsen D. W., Gao Y., DeLeo F. R., Quinn M. T. (2007). Role of NF-*κ*B in transcriptional regulation of the phagocyte NADPH oxidase by tumor necrosis factor-*α*. *Journal of Leukocyte Biology*.

[8] Suzuki Y., Hattori K., Hamanaka J. (2012). Pharmacological inhibition of TLR4-NOX4 signal protects against neuronal death in transient focal ischemia. *Scientific Reports*.

[9] Lee I.-T., Shih R.-H., Lin C.-C., Chen J.-T., Yang C.-M. (2012). Role of TLR4/NADPH oxidase/ROS-activated p38 MAPK in VCAM-1 expression induced by lipopolysaccharide in human renal mesangial cells. *Cell Communication and Signaling*.

[10] Choi Y. Y., Kim M. H., Han J. M. (2014). The anti-inflammatory potential of Cortex Phellodendron in vivo and in vitro: down-regulation of NO and iNOS through suppression of NF-*κ*B and MAPK activation. *International Immunopharmacology*.

[11] Ohtsuki M., Nomura T., Morimoto S.-I. (2005). Suppressed expression of GTP cyclohydrolase I mRNA and accelerated expression of inducible nitric oxide synthase mRNA in endomyocardial biopsy specimens from patients with dilated cardiomyopathy. *Clinica Chimica Acta*.

[12] Lee J.-Y., Yang H. J., Kim J.-M., Jeon B. S. (2013). Novel *GCH-1* mutations and unusual long-lasting dyskinesias in Korean families with dopa-responsive dystonia. *Parkinsonism & Related Disorders*.

[13] Jilling T., Simon D., Lu J. (2006). The roles of bacteria and TLR4 in rat and murine models of necrotizing enterocolitis. *The Journal of Immunology*.

[14] Faure E., Equils O., Sieling P. A. (2000). Bacterial lipopolysaccharide activates NF-*κ*B through toll-like receptor 4 (TLR-4) in cultured human dermal endothelial cells. Differential expression of TLR-4 and TLR-2 in endothelial cells. *Journal of Biological Chemistry*.

[15] Seki E., Tsutsui H., Nakano H. (2001). Lipopolysaccharide-induced IL-18 secretion from murine Kupffer cells independently of myeloid differentiation factor 88 that is critically involved in induction of production of IL-12 and IL-1*β*. *The Journal of Immunology*.

[16] Haynes L. M., Moore D. D., Kurt-Jones E. A., Finberg R. W., Anderson L. J., Tripp R. A. (2001). Involvement of Toll-like receptor 4 in innate immunity to respiratory syncytial virus. *Journal of Virology*.

[17] Roy M.-F., Larivière L., Wilkinson R., Tam M., Stevenson M. M., Malo D. (2006). Incremental expression of Tlr4 correlates with mouse resistance to *Salmonella* infection and fine regulation of relevant immune genes. *Genes and Immunity*.

[18] Deng S., Wu Q., Yu K. (2012). Changes in the relative inflammatory responses in sheep cells overexpressing of toll-like receptor 4 when stimulated with LPS. *PLoS ONE*.

[19] Bihl F., Salez L., Beaubier M. (2003). Overexpression of toll-like receptor 4 amplifies the host response to lipopolysaccharide and provides a survival advantage in transgenic mice. *The Journal of Immunology*.

[20] Yu J. H., Kim H. (2014). Oxidative stress and inflammatory signaling in cerulein pancreatitis. *World Journal of Gastroenterology*.

[21] Gunjaca I., Zunic J., Gunjaca M., Kovac Z. (2012). Circulating cytokine levels in acute pancreatitis—model of SIRS/CARS can help in the clinical assessment of disease severity. *Inflammation*.

[22] Szajnik M., Szczepanski M. J., Czystowska M. (2009). TLR4 signaling induced by lipopolysaccharide or paclitaxel regulates tumor survival and chemoresistance in ovarian cancer. *Oncogene*.

[23] Zhao J., Zhao S., Zhou G. (2011). Altered biliary epithelial cell and monocyte responses to lipopolysaccharide as a TLR ligand in patients with primary biliary cirrhosis. *Scandinavian Journal of Gastroenterology*.

[24] De Loera-Rodriguez C., Delgado-Rizo V., Alvarado-Navarro A., Agraz-Cibrian J., Segura-Ortega J. E., Fafutis-Morris M. (2014). Over-expression of TLR4-CD14, pro-inflammatory cytokines, metabolic markers and NEFAs in obese non-diabetic Mexicans. *Journal of Inflammation*.

[25] Wittebole X., Castanares-Zapatero D., Laterre P. F. (2010). Toll-like receptor 4 modulation as a strategy to treat sepsis. *Mediators of Inflammation*.

[26] LaRue H., Ayari C., Bergeron A., Fradet Y. (2013). Toll-like receptors in urothelial cells—targets for cancer immunotherapy. *Nature Reviews Urology*.

[27] Kim J. J., Sears D. D. (2010). TLR4 and insulin resistance. *Gastroenterology Research and Practice*.

[28] Cho H.-Y., Kleeberger S. R. (2007). Genetic mechanisms of susceptibility to oxidative lung injury in mice. *Free Radical Biology & Medicine*.

[29] Nackiewicz D., Dan M., He W. (2014). TLR2/6 and TLR4-activated macrophages contribute to islet inflammation and impair beta cell insulin gene expression via IL-1 and IL-6. *Diabetologia*.

[30] Heo S.-K., Yun H.-J., Noh E.-K., Park W.-H., Park S.-D. (2008). LPS induces inflammatory responses in human aortic vascular smooth muscle cells via Toll-like receptor 4 expression and nitric oxide production. *Immunology Letters*.

[31] Taylor B. S., de Vera M. E., Ganster R. W. (1998). Multiple NF-*κ*B enhancer elements regulate cytokine induction of the human inducible nitric oxide synthase gene. *The Journal of Biological Chemistry*.

[32] Dikalova A. E., Góngora M. C., Harrison D. G., Lambeth J. D., Dikalov S., Griendling K. K. (2010). Upregulation of Nox1 in vascular smooth muscle leads to impaired endothelium-dependent relaxation via eNOS uncoupling. *The American Journal of Physiology: Heart and Circulatory Physiology*.

[33] Xia N., Daiber A., Habermeier A. (2010). Resveratrol reverses endothelial nitric-oxide synthase uncoupling in apolipoprotein E knockout mice. *Journal of Pharmacology and Experimental Therapeutics*.

[34] Gopalakrishnan B., Nash K. M., Velayutham M., Villamena F. A. (2012). Detection of nitric oxide and superoxide radical anion by electron paramagnetic resonance spectroscopy from cells using spin traps. *Journal of Visualized Experiments*.

[35] Zhang X., Shan P., Qureshi S. (2005). Cutting edge: TLR4 deficiency confers susceptibility to lethal oxidant lung injury. *Journal of Immunology*.

[36] Bautista A. P., Spitzer J. J. (1994). Inhibition of nitric oxide formation in vivo enhances superoxide release by the perfused liver. *The American Journal of Physiology: Gastrointestinal and Liver Physiology*.

[37] Pryor W. A., Squadrito G. L. (1995). The chemistry of peroxynitrite: a product from the reaction of nitric oxide with superoxide. *The American Journal of Physiology—Lung Cellular and Molecular Physiology*.

[38] Moens A. L., Kass D. A. (2007). Therapeutic potential of tetrahydrobiopterin for treating vascular and cardiac disease. *Journal of Cardiovascular Pharmacology*.

[39] Schmidt T. S., McNeill E., Douglas G. (2010). Tetrahydrobiopterin supplementation reduces atherosclerosis and vascular inflammation in apolipoprotein E-knockout mice. *Clinical Science*.

[40] Sakai K., Suzuki H., Oda H. (2006). Phosphoinositide 3-kinase in nitric oxide synthesis in macrophage: critical dimerization of inducible nitric-oxide synthase. *The Journal of Biological Chemistry*.

[41] Gross S. S., Levi R. (1992). Tetrahydrobiopterin synthesis. An absolute requirement for cytokine-induced nitric oxide generation by vascular smooth muscle. *Journal of Biological Chemistry*.

[42] Wang W., Zolty E., Falk S. (2008). Endotoxemia-related acute kidney injury in transgenic mice with endothelial overexpression of GTP cyclohydrolase-1. *The American Journal of Physiology—Renal Physiology*.

[43] Geller D. A., di Silvio M., Billiar T. R., Hatakeyama K. (2000). GTP cyclohydrolase I is coinduced in hepatocytes stimulated to produce nitric oxide. *Biochemical and Biophysical Research Communications*.

[44] Cai S., Alp N. J., McDonald D. (2002). GTP cyclohydrolase I gene transfer augments intracellular tetrahydrobiopterin in human endothelial cells: effects on nitric oxide synthase activity, protein levels and dimerisation. *Cardiovascular Research*.

[45] Alp N. J., Mussa S., Khoo J. (2003). Tetrahydrobiopterin-dependent preservation of nitric oxide-mediated endothelial function in diabetes by targeted transgenic GTP-cyclohydrolase I overexpression. *The Journal of Clinical Investigation*.

